# ORAI Calcium Channels: Regulation, Function, Pharmacology, and Therapeutic Targets

**DOI:** 10.3390/ph16020162

**Published:** 2023-01-22

**Authors:** Hussein N. Rubaiy

**Affiliations:** Department of Laboratory Medicine, Division of Clinical Pharmacology, Karolinska Institute and Karolinska University Hospital, C1:68, 141 86 Stockholm, Sweden; hussein.rubaiy@ki.se

**Keywords:** ORAI, STIM, ion channel, pharmacology, calcium channel, therapeutic targets

## Abstract

The changes in intracellular free calcium (Ca^2+^) levels are one of the most widely regulators of cell function; therefore, calcium as a universal intracellular mediator is involved in very important human diseases and disorders. In many cells, Ca^2+^ inflow is mediated by store-operated calcium channels, and it is recognized that the store-operated calcium entry (SOCE) is mediated by the two partners: the pore-forming proteins Orai (Orai1-3) and the calcium store sensor, stromal interaction molecule (STIM1-2). Importantly, the Orai/STIM channels are involved in crucial cell signalling processes such as growth factors, neurotransmitters, and cytokines via interaction with protein tyrosine kinase coupled receptors and G protein-coupled receptors. Therefore, in recent years, the issue of Orai/STIM channels as a drug target in human diseases has received considerable attention. This review summarizes and highlights our current knowledge of the Orai/STIM channels in human diseases and disorders, including immunodeficiency, myopathy, tubular aggregate, Stormorken syndrome, York platelet syndrome, cardiovascular and metabolic disorders, and cancers, as well as suggesting these channels as drug targets for pharmacological therapeutic intervention. Moreover, this work will also focus on the pharmacological modulators of Orai/STIM channel complexes. Together, our thoughtful of the biology and physiology of the Orai/STIM channels have grown remarkably during the past three decades, and the next important milestone in the field of store-operated calcium entry will be to identify potent and selective small molecules as a therapeutic agent with the purpose to target human diseases and disorders for patient benefit.

## 1. Introduction

The hint of SOCE that describes how Ca^2+^ inflow across the plasma membrane is triggered by the depletion of endoplasmic reticulum (ER) Ca^2+^ reserves was first introduced by [1] about three decades ago. Today, the concept of SOCE is well-established and universal in various mammalian cell types. This unique mechanism of Ca^2+^ entry controls the basal calcium, refills intracellular Ca^2+^ stores, and performs an extensive variety of specific activities. Therefore, SOCE is involved in very important physiological and pathological processes [2]. These processes comprise gene expression, proliferation, secretion, and metabolism, as well as cell growth. Furthermore, many studies have reported that SOCE is involved in various human diseases, such as allergies, cancer, some types of immunodeficiency and autoimmunity disorders, inflammatory bowel disease, and—recently by our lab—diabetic nephropathy [2,3].

### 1.1. Membrane Topology Structure of the ORAI/STIM Channel Complexes

**Figure 1:**
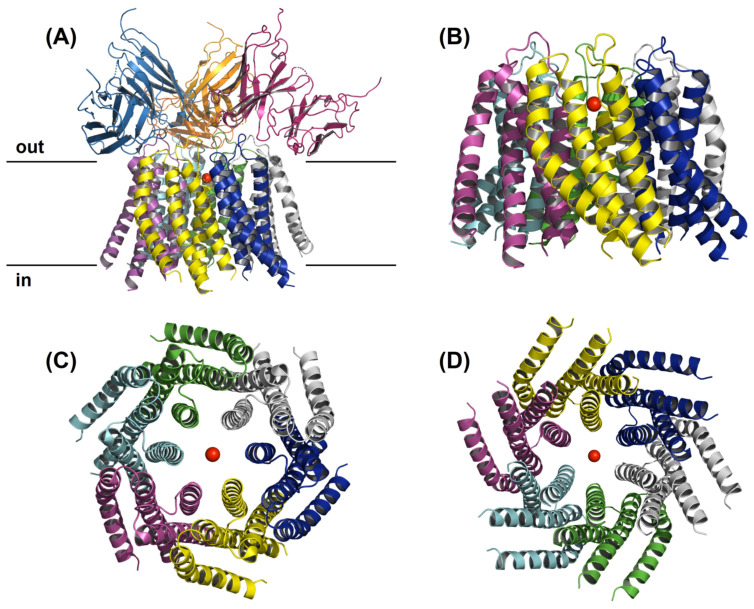
The hexameric Orai channel structure. The figure illustrates a side view of the overall structure of Orai channel complex from the perspective of the membrane (**A**), a side view of just the four TMs (**B**), a top view (**C**), and intracellular view (**D**). Each subunit contains four transmembrane helices as represented in different colours. The red ball in the centre of the pore represents the Ca^2+^ ion.

Ion channels are homomeric or heteromeric protein subunit assemblies to form ion conduction pathways through cell membranes, which are otherwise very hydrophobic. Moreover, channel dimers, trimers, tetramers, pentamers, and hexamers have all been depicted [4]. Following the discovery of genes encoding Orai family members, scientists began using biochemical, functional, and imaging approaches to conclude the stoichiometric composition of the Orai channel pore. Tandem dimeric, trimeric, or tetrameric Orai1 constructs with or without pore-blocking dominant-negative mutations were co-expressed with STIM1 to investigate Orai channel stoichiometry [5]. In the past, the Orai channel was thought to be tetrameric; however, based on crystallographic and electrophysiological investigations, it is now believed to be a hexamer of Orai subunits (Figure 1), [6,7,8,9].

In 2012, Hou et al. solved the X-ray crystal structure of a closed conformation of Drosophila melanogaster Orai, which provided the first glimpse of the channel’s molecular architecture [7]. The structure was resolved at 3.35 Å resolution and revealed that the channel is made up of a hexamer of Orai subunits instead of a tetrameric assembly, as had been predicted at the time [7]. Moreover, other groups confirmed that human Orai1 channels can likewise assemble and function as hexamers [10,11]. Today, it is well established that the channel is composed of six subunits, as shown in Figure 1, and each subunit comprises four transmembrane helices, TMHs (M1, M2, M3, and M4). Figure 1 illustrates the hexameric Orai channel structure and demonstrates that each subunit contains four transmembrane helices, as represented in different colours. Bioinformatics software Pymol was used to create structures based on known protein sequences and work from Hou et al. [12]. In more detail, the channel contains a single ion conduction pore along a central sixfold axis of symmetry, which in a cellular setting would be vertical to the membrane. Six M1 helices, one from each subunit, constitute the walls of the ion pore, which measures around 55 Å in length and is narrow in the closed conformation [12].

In brief, Yamashita et al. described how the pore opens in response to STIM1 binding [13]. In more detail, this group reported that V102 and F99, which are two rings of the pore-lining residues working as partners to form a hydrophobic gate. Moreover, mutations of residues to polar amino acids create channels with leaky gates operating ions in resting state. Pore helix rotation causes STIM1-mediated channel activation, which moves the F99 residues away from the pore axis, resulting in increased pore hydration. This enables ions to flow through the V102-F99 hydrophobic band. This rotation discloses the dynamic coupling between Ca^2+^ release-activated Ca^2+^ (CRAC) channel gating and ion selectivity. This hydrophobic gating mechanism plays a role in CRAC channel function, pharmacology, and disease-causing mutations [13].

Tiffner and colleagues very recently identified that isoform-specific nonconserved gating checkpoints in transmembrane domain 3 (TM3) of Orai1 and Orai3 maintain a closed state and an opening permissive channel conformation, whereas global conformational TM movements are required for pore opening of both channels [14]. That indicates that TM3 controls the pore opening in an isoform-specific manner for both Orai1 and Orai3 proteins [14].

### 1.2. Activation and Regulation of ORAI/STIM Channel Complexes

Calcium (Ca^2+^) ion is a universal second messenger that controls wide diversity and very critical functions in almost all cell types. These cellular functions involve muscle contraction, neuronal transmission, cell migration, cell growth, gene transcription, and cell death. Therefore, dysregulation of Ca^2+^ signals is linked to major diseases in humans such as cardiovascular and neurological diseases, cancer, and others [15]. Ion channels are pore-forming proteins that participate and play crucial roles in very vital physiological and pathological processes such as neuronal signalling and cardiac excitability; as a result, they serve as therapeutic drug targets such as in diabetes mellitus [4,16].

It is well established that the store-operated calcium entry is mediated by the two partners, the pore-forming proteins Orai (Orai1-3) and the calcium store sensor, stromal interaction molecule (STIM1-2). Briefly, in numerous mammal cells, the store-operated (CRAC) channels function as a vital path for Ca^2+^ entry and defects in CRAC channel utility are linked with severe human diseases such as immunodeficiency and autoimmunity [2].

Various extracellular signals can trigger GPCRs and/or Receptor Tyrosine Kinases (RTKs) that are localized on the cell membrane. This results from Phospholipase C-mediated enzymatic hydrolysis of Phosphatidyl inositol 4,5-bis phosphate (PIP2) and generation of the intracellular messenger inositol-1,4,5-triphosphate (IP3). Inositol-1,4,5-triphosphate receptor (IP3R): IP3 binds to the IP3R, which is largely found in the ER [17]. In brief, the IP3R is a ligand-activated Ca^2+^ channel. Following a ligand binding to the IP3R, Ca^2+^ flows from the ER to the cytosol along a favourable concentration gradient. Stromal interaction molecules (STIM) on the ER membrane sense the drop in ER Ca^2+^ because they are near the ER membrane [18]. STIM proteins operate as sensors for Ca^2+^ stored in the ER lumen and subsequently translocate into the ER-plasma membrane junctions, where they bind to and activate Orai channels. In other words, to generate the Ca^2+^-selective, CRAC current, the Orai channels are activated directly by STIM proteins, consequent to ER Ca^2+^ store depletion [19]. Notably, STIM proteins have a unique N-terminal calcium sensing domain that allows them to respond to changes in the ER luminal calcium concentration. This domain provides STIM proteins the ability to sense calcium levels in the ER [20]. STIM1 directly gates and activates the Orai1 after STIM1 binds to the C- and N- terminal cytosolic domains of Orai1. Notably, CRAC-activation domain, recognized as the STIM-Orai activating region (SOAR), contains ~100 amino acids and is essential for the activation of the channel [21,22].

As an example, the SOCE in VSMCs is mediated by the Orai proteins. Activation of Orai1 channels is induced by Ca^2+^ release from the ER via the IP3 receptor, which leads in store depletion and the formation of STIM1 oligomers. Somatic cell migration and proliferation (SOCE) mediated by Orai1 are related with the stimulation of the nuclear factor of the activated T-cell (NFAT), which increases the proliferation and migration of VSMCs. Additionally, Ca^2+^ influx through Orai1 stimulates mitochondrial Ca^2+^ uptake through the mitochondrial Ca^2+^ uniporter [23].

The involvement of classical transient receptor potential TRPC1, TRPC4, and TRPC5 (TRPC1/4/5) channels in SOCE has been controversial in the Ca^2+^ channel field [24]. Remarkably, the Pico145 blocker, which is a potent and selective inhibitor of TRPC1/4/5 channels, failed to produce any noticeable effect on the SOCE that was mediated by Orai1 [25,26,27,28,29]. These findings lead us to believe that the contribution of TRPC1/4/5 channels to store-operated Ca^2+^ entry should be disregarded in A498, HEK 293, and HUVEC cells [25,26,27,28,29].

## 2. ORAI/STIM Channel Complexes in Human Diseases

In recent years, the issue of Orai/STIM channels as a drug target in human diseases has received considerable attention. It is well-established that the store-operated calcium entry is mediated by the two partners: the pore-forming proteins Orai (Orai1-3) and the calcium store sensor, stromal interaction molecule (STIM1-2). Briefly, in numerous animal cells, the store-operated Ca^2+^ release-activated Ca^2+^ (CRAC) channels function as a vital path for Ca^2+^ entry, and defects in the CRAC channel event are linked with severe human diseases such as immunodeficiency and autoimmunity [30].

The intention of this section is to review, focus, and discuss the involvement of Orai/STIM channels in different and important human diseases and disorders such as immunodeficiency, myopathy, tubular aggregate, Stormorken syndrome, York platelet syndrome, cancers, and cardiovascular and metabolic disorders.

### 2.1. Immunodeficiency

Nearly two decades ago, Feske and his colleagues by applying two unbiased genetic methods reported a modified linkage analysis to categorize the gene mutated in the severe combined immune deficiency (SCID) patients, and a genome-wide RNA interference (RNAi) screen identified that a mutation in Orai1, R91W, produces immune deficiency by abolishing CRAC channel function [30]. This mutation denotes a nonfunctional CRAC channels as a result of a defect in gating than in an interaction with full-length STIM1 [31].

In addition, Orai1 is a vital element or mechanism of the CRAC channel complex [32]. Stimulation of immune cells by antigens triggers Ca^2+^ entry through CRAC channels, contributing to the immune response to pathogens by triggering the NFAT. The same group reported earlier that deficiency in SOCE and CRAC channel function is associated as hereditary to SCID syndrome in humans [32].

Immunodeficient patients with Orai1 deficiency suffer from persistent critical infections with viral bacterial, mycobacterial and fungal pathogens, which leads to pneumonia, meningitis, enteritis, GI candidiasis and sepsis due to a number of mutations, e.g., G98R and A103E, two loss-of-function mutations that abolish SOCE (Table 1) [33,34,35]. In patients lacking functional Orai expression, the immunodeficiency is dominated by a defect in T-cell activation and possibly B- and NK-cell activation, whereas lymphocyte development is normal [33]. The clinical symptoms resemble those found in patients with severe combined immunodeficiency. A majority of all patients with Orai1 deficiency exhibit increased susceptibility to a number of herpes virus infections.

### 2.2. Myopathy

Recently, as SOCE is the major mechanism that regulates the Ca^2+^ homeostasis that is facilitated by the two partners STIM1 and Orai1, Böhm et al. reported that Orai1 mutations, which have distinct channel gating defects, are associated beside Stormorken Syndrome and tubular aggregate myopathy (TAM) [36]. Böhm et al. suggested in their study on Orai1 channels that TAM and Stormorken syndrome are varieties of a similar disease, as their data stipulated a mutation-dependent pathomechanism and a genotype/phenotype correlation as the Orai1 mutations, which are related to the furthermost severe indications, producing the most significant functional influence on the cells (Table 1) [37]. The two syndromes are involved in a spectrum of clinical manifestations, including muscle weakness, skin disorder ichthyhosis, eye disorder miosis, blood disorder thrombocytopenia, and learning disorder dyslexia.

### 2.3. Tubular Aggregate Myopathy

Tubular aggregate myopathy (TAM) is a rare form of myopathy that affects the skeletal muscles. Muscle biopsies taken from TAM patients show densely packed membrane tubules, and symptoms include weakness, cramps, and muscle pains. TAM commonly affects the proximal muscles of the lower limbs, causing loss of mobility, including difficulties running and climbing stairs (Table 1) [36].

TAM and Stormorken syndrome are both caused by gain-of-function mutations. This type of mutation results from an alteration of gene products in which a new molecular function or new gene expression pattern are formed. The gain-of-function mutations in STIM1 or Orai1 and additional mutations in CASQ1 cause excessive Ca^2+^, leading to the two syndromes. These three genes are involved in the control of Ca^2+^ homeostasis [36]. All STIM1 mutations that are involved in TAM have shown to be missense mutations located in the EF hand, helix–loop–helix structural domain. It is suggested that these mutations prompt the constitutive ORAI1 activation and generate excessive Ca^2+^ entry into muscle cells [38]. Mutation STIM1-I484R has been located not in the EF hand but in STIM1′s cytoplasmic C-terminal inhibitory domain (CTID). It has been reported that cells transfected with STIM-I484R showed an aggregation-like appearance of STIM1 and reduced Ca^2+^ influx [38]. Further on, it was determined that frameshift STIM-I484R mutation induces a gain in CRAC channel function due to the loss of critical inhibitory C-terminal domains. This blocks STIM1 to bind to ORAI1, which then enables STIM1 trapping by microtubules and interferes slow Ca^2+^-dependent inhibition. This might provide a mechanistic explanation of why TAM patients with this mutation receive muscular defects [39].

ORAI-G98S, -V107M, and T184M are three other mutations of ORAI1 that are all involved in TAM. G98S and V107M mutations cause constitutive channel opening independently of STIM, while the third mutation, T184M, which is not located in the channel pore, overacts depending on STIM presence. This suggests that these mutations located in different ORAI1 transmembrane domains seem to involve different pathomechanisms revealing a genotype–phenotype correlation [37].

ORAI1-G98S is a heterozygous missense mutation that causes TAM with hypocalcemia due to constitutive activation of CRAC channels. A study involving ORAI1-G98S and other constitutive activity ORAI1 mutations—V107M, L138F, T184M, and P245L—showed when ORAI blocker GSK-7975A was applied the G98S mutant was resistant to inhibition. This is while GSK-7975A blocked the constitutive activity of the other mutants, indicating that it could possibly be used in therapy for TAM patients [40,41].

STIM mutations also play a role in TAM, e.g., EF hand mutation STIM1-D84G, which causes constitutive Ca^2+^ influx present in patients with nonsyndromic TAM. Interestingly, a study involving mice with STIM1-D84G developed a bleeding disorder with thrombocytopenia, a condition when blood platelet count is too low. Moreover, mutation STIM-H109N causes somewhat different disease symptoms, such as either predominantly postexercise fatigability and episodic double vision or weakness in lower limb muscles and contractures [42].

### 2.4. Stormorken Syndrome

Tubular aggregate myopathy is also present in a rare multiorgan disease, Stormorken syndrome, and this has led to discussions about considering the two as part of one clinical continuum. Stormorken syndrome is caused by autosomal dominant mutations, and its clinical features include miosis (permanent constriction of the eye pupils), thrombocytopenia (low blood platelet count), ichthyosis (widespread and persistent thick, dry, “fish-scale” skin), hyposplenism (physiological loss of spleen function), dyslexia, and short stature [43].

Stormorken syndrome is clinically fully expressed in patients who are affected by mutations of STIM and/or ORAI. STIM1-R304W mutation in functional studies showed a possible mutational influence on Orai1 channel deactivation; however, the specific pathomechanism of the syndrome is yet to be identified (Table 1) [44]. R304W in STIM1 activates allele more strongly than mutation P245L in ORAI1, which prevents slow Ca^2+^-dependent inactivation, however, does not cause a constitutively active CRAC channel. ORAI1-P245L is located with the fourth transmembrane helix, M4, of ORAI1, and it acts as a gain-of-function mutation [44].

Currently, there is no treatment for TAM and Stormorken syndrome; however, it has been shown that Ca^2+^ balance can be manipulated by chemical compounds. It has been indicated that reducing excessive Ca^2+^ influx through inhibition of CRAC channels could be a possible therapeutic approach in the treatment. Further studies are needed to determine if an imbalance of other ions could be a factor to the development of Stormorken syndrome and TAM [36].

### 2.5. York Platelet Syndrome

Within the same clinical continuum as TAM and Stormorken syndrome is York platelet syndrome (YPS). Clinical studies have shown that its muscular and nonmuscular phenotypes are consistent with the two other syndromes [45]. York platelet syndrome is a Ca^2+^ channelopathy caused by gain-of-function in STIM1 through heterozygous mutations, including c.343A > T and c.910C > T [45]. YPS represents a rare genetic form of the blood disorder thrombocytopenia, with striking ultrastructural platelet abnormalities such as giant electron opaque organelles and massive, multilayered target bodies, as well as a deficiency of platelet Ca^2+^ storage in delta granules.

A majority of patients suffering from YPS have a greater tendency to bleeding with epistaxis (nose bleeding), bruising, and postpartum haemorrhage. Notably, YPS and Stormorken syndrome are associated with same types of symptoms, and some suggest that the two may be the same clinical condition but with different names (Table 1) [46].

### 2.6. Cardiovascular and Metabolic Disorders

Calcium ions’ involvement in cardiovascular diseases is well known and was first introduced by Putney [1]. A number of studies have shown that vascular illnesses are significantly influenced by the dysregulation of the SOCE pathway. In most vascular disorders, there is an increased presence of STIM1, Orai1, and Orai3, which results in dysregulation of cellular Ca^2+^ homeostasis, which in turn leads to vascular pathologies. It has been discovered that STIM and Orai proteins are key molecular players of the SOCE pathway [47]. Because of these breakthroughs, the roles of STIM1 and Orai1 in mediating the increase in Ca^2+^ platelet concentration and thrombosis are now better understood.

Grosse et al. and Ahmad et al. have suggested that STIM1 is involved in thrombus formation and its stability. In a separate investigation, Xia et al. stated that an increased STIM1 function in patients with type 2 diabetic patients could be a contributing factor to their high tendency to thrombosis [48,49,50,51].

STIM1 and Orai1 also play a role in atherosclerosis, with studies showing that an increased presence SOCE in VSMCs prior to development of atherosclerosis plaques [52]. According to many studies, high-fat diets increase SOCE and STIM1 expression in coronary smooth cells, which is associated with enhanced atherosclerosis lesions [47]. It has also been shown that STIM1 is linked to the regulation of the endothelial barrier function and progenitor endothelial cells functions in atherosclerotic lesions [53]. STIM1 plays a key role in GPCR-activated endothelial barrier function alterations independent of Ca^2+^ and Orai1 [53]. A separate study has reported the significant role of Orai1 in atherosclerosis by its regulation of both vascular inflammation and foam cell formation [54]. In conclusion, these studies propose that STIM1 and Orai1 are involved in atherosclerosis plaque development. STIM and Orai proteins also play a critical role in pulmonary and vascular hypertension, as studies show a very strong association between hypertension and the augmented presence of the two proteins [55].

### 2.7. Cancers

Growing evidence has shown that numerous cancer types, including liver, lung, ovarian, breast, colon, and gastric cancer, as well as multiple myeloma, manifest increased SOCE and augmented expression of STIM1 or Orai1. Additionally, it has been claimed that SOCE could play a role in the oncogenic pathway, since its blockage, which can be achieved through the knockdown of STIM1 or Orai1, prevents the proliferation and spread of cancer cells [56,57,58,59,60,61]. It is suggested that SOCE stimulates the proliferation and metastasis of cancer cells, enhances tumour angiogenesis and the formation of a tumour-promoting inflammatory environment [58]. A study by Chen et al. demonstrated that STIM1 is able to control the production of vascular endothelial growth factor (VEGF) in cervical cancer by inhibiting STIM1, and the ability to govern the growth of blood vessels and tumours could be impaired [62]. Upregulation of STIM1 in primary cervical tumours was strongly linked with larger tumour size and elevated lymph node metastasis, and therefore poorer clinical outcomes [62]. A study involving immunostaining of overexpressed STIM1 and STIM2 in human cervical cancer revealed that STIM1 is the main endoplasmic reticulum Ca^2+^-sensing molecule found in the invasive tumour front, indicating that STIM1 plays a role in both tumour growth and invasion. This is while STIM2 is mainly associated with tumour growth (Table 1) [63]. In multiple myeloma patients, overexpression of STIM1/Orai1 was closely linked with shorter progression-free survival (Table 1) [60]. This was also observed in individuals with oesophageal squamous cell carcinoma, as increased expression of Orai1 in tumour tissue was associated with a worse prognosis overall and recurrence-free survival [64].

Schild et al. developed novel effective inhibitors: several monosubstituted and amino acid analogues of 2-APB and SOCE using a fluorometric imaging plate reader (FLIPR) assay format in MDA-MB-231 cells [65]. Compared to 2-APB, the compound series of monohalogenated 2-APB derivatives demonstrated potential inhibitory efficacy. Analogues *m*-Cl-2APB, *m*-Br-2APB, *m*-I-2APB, and *p*-I-2APB blocked SOCE at five times lower concentrations than 2-APB, and notably, 2-APB increased SOCE at this low concentration [65].

Recently, more and more evidence is indicating that Orai1′s two homologs, Orai2 and Orai3, play a role in the development of different types of cancer such as lung, breast, prostate, and leukaemia [66]. Studies have shown that Orai3 plays an essential role in lung cancer, with one study revealing that immunostaining of Orai3 was overexpressed in lung cancer tissues compared to nontumorous ones, and was linked to a high tumour grade [67]. In a separate study, Orai3 immunostaining showed a correlation between overexpressed Orai3 and the aggressiveness of lung adenocarcinoma [68].

The most commonly occurring cancer among women is breast cancer. In cancer cells, SOCE channels are extensively remodelled and are involved in the development and support of different cancer hallmarks. In all investigated breast cancer cell subtypes, Orai1 is overexpressed, and its cell biology is strongly dependent on CRAC channels. It has also been shown that Orai3 is selectively overexpressed in luminal A breast cancer cells where both SOCE and the development of different cancer features such as cell migration and proliferation are greatly dependent on Orai3. The role of Orai2 in breast cells was not determined until recently, when Sanchez-Collado et al. suggested that Orai2 is needed for cell cycle progression and apoptosis resistance in cells with high Orai2 expression [66]. The group indicated that based on their findings, Orai2 plays a crucial role in the pathophysiology of breast cancer subtypes with an elevated Orai2 expression profile [66]. Furthermore, Sanchez-Collado et al. suggested that Orai2′s role in cell cycle progression could be one factor contributing to chemoresistance in breast cancer cells [66].

Prostate cancer is the third most lethal tumour among men, with more than 500,000 new cases every year. Orai1, 2, and 3 are all expressed in normal and cancerous epithelial prostatic cells. It has been determined that in prostate cancer, remodelling of channel-forming Orai proteins induces an oncogenic switch [69]. It has also been shown that in prostate cancer samples, the relative expression of the Orai3 transcript is highly elevated in comparison to paired-match noncancerous tissue [66].

With the determination of the roles of Orai and STIM in cancer, studies involving the pharmacological inhibition of SOCE or downregulation of STIM1 have been revealed to enhance apoptosis induced by cisplatin in non-small cell lung cancer cells, and Orai1/STIM1 are associated with a protective antiapoptotic role in ovary carcinoma cells [70]. In cancer treatment, there is a great potential in inhibition of SOCE; however, its usage should be with caution due to the abundant expression of STIM and Orai proteins in the human body and their vital roles in the immune system. Therefore, one focus should be on developing SOCE modulators that target specific tumour cells or tumour-associated vascular endothelial cells by designing assays in the presence of both cancer and immune cells. Another focus should be to develop therapeutic approaches that target cancer cell-specific mechanisms of SOCE activation [71].

### 2.8. Nervous System

Orai and STIM proteins are present in normal brain function; however, they are also involved in neurological disorders such as Alzheimer’s disease, epilepsy, brain trauma, and cerebral infarction. The role of Orai proteins in neuronal calcium signalling is yet to be determined.

It has, however, been reported that STIM2 is abundantly active in hippocampal and cortical neurons, which activates neuronal SOCE. It is involved in regulating steady-state activity of phosphorylated Ca^2+^/calmodulin-dependent protein kinase II (CaMKII) and consequently stabilizes mushroom spines in hippocampal neurons. It is suggested that weakened STIM activity and loss of mushroom spines may play a role in memory deficiency and in Alzheimer’s disease. Regarding epilepsy, brain trauma, and cerebral infarction, STIM2 could possibly be a potential target for the treatment of glutamate-induced excitotoxicity, which leads to loss of neuronal function and cell death [72].

### 2.9. Kidney

Orai channels are also found in human kidney’s proximal tubules. Dysfunction of these proteins in the proximal tubules causes an absorption decrease in albumin by its cells and leads to proteinuria [2].

Furthermore, store-operated Ca^2+^ channel Orai1 activity plays a strong role in lymphocytes, especially TH17 cells, which regulate the severity of both acute injury and chronic kidney disease (Table 1) [73]. A study conducted by Basile revealed that inhibition Orai1 diminishes both acute and chronic kidney injury in rodent models [73]. Orai1+ cells are overexpressed in peripheral blood of patients suffering acute kidney injury, suggesting that Orai1 could possibly be a target in the acute kidney injury to chronic kidney disease transition. It is yet to be determined if inhibition of the endothelial STIM1/Orai1 pathway prevents kidney injury and where such activity plays a role in inflammation and/or haemodynamics [73].

In a separate study, Mai et al. revealed that the Orai1 calcium channel plays a role in renal fibrosis induced by a high-fat diet and by unilateral ureteral obstruction [74]. According to their results, Orai1-mediated Ca^2+^ entry is involved in the progression of renal fibrosis, which might lead to end-stage renal disease; however, the molecular mechanisms remain undetermined. Mai et al. suggest that blocking the Orai1 Ca^2+^ channel could possibly be a therapeutic option to curb the progress of kidney fibrosis [74].

## 3. Pharmacology of Store-Operated Calcium Entry Channels

Recent developments in the function of Orai/STIM channels in human physiology and pathology have led to a renewed interest in the identification of small molecules as modulators or pharmacological agents to target these channels. However, major problems in the field of the Orai/STIM channel pharmacology have been the identification of selective subtype-specific and potent modulators of these channels.

### 3.1. Activators of ORAI/STIM Channel Complexes

**Figure 2:**
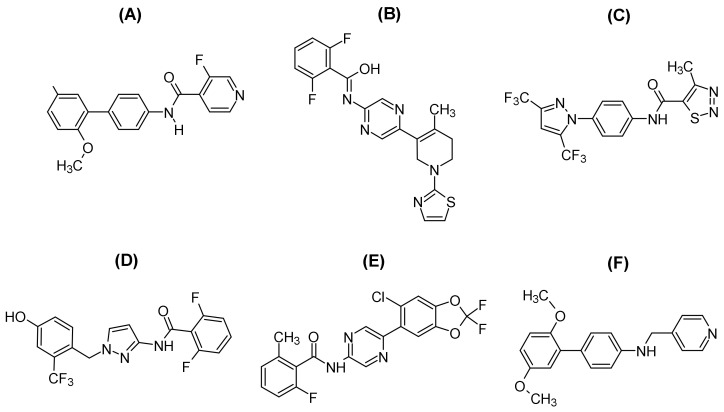
The chemical structure of Orai/STIM channel blockers. (**A**) Synta-66, (**B**) RO2959, (**C**) BTP2, (**D**) GSK-7975A, (**E**) CM4620, (**F**) JPIII.

The activation process of Orai/STIM channels is very complex; therefore, several pharmacological tools can be developed to modulate these channels’ activity [76]. The Orai/STIM channels can be activated by phytohaemagglutinin (PHA), IP3, cyclopiazonic acid (CPA), ionomycin, BAPTA, EGTA, and thapsigargin (TG) [24,77]. In a nutshell, these substances induces ER Ca^2+^ release through IP3R, which blocks SERCA to make ER Ca^2+^ depletion, and which induces a passive ER Ca^2+^ depletion [78]. Moreover, conventionally nonspecific and low-potency blockers such as 2-APB, La^3+^, and SKF96365 have regularly been used to study the physiological function of Orai/STIM channels in different cells and tissues [79]. However, selective and potent pharmacological tools that can be used to assess the physiological and pathological role of Orai/STIM channels or develop as therapeutics drugs have been absent (Figure 2). Therefore, the inspiration to discover a novel selective and potent antagonist or agonist has been a necessity in recent years.

### 3.2. Thapsigargin

Thapsigargin, often known as TG, was first extracted from the roots of the Mediterranean umbelliferous plant *Thapsia garganica* in order to investigate the nature of its skin-irritant component. The *Thapsia L.* species, also known as deadly carrots, have been utilized in traditional medicine for thousands of years in the Mediterranean region. Furthermore, TG treatment of mammalian cells was demonstrated to result in increased calcium levels in the cytoplasm, and TG was established as blocker of the sarcoendoplasmic reticulum Ca^2+^-ATPase (SERCA) in 1990 [80]. TG has a unique chemical structure; in addition, the primary and most significant pharmacological effect of TG is its strong SERCA pump inhibition. Moreover, this effect is substantial at concentrations below one nanomolar [80,81].

### 3.3. 2T and 3G

Riva and colleagues, based on the Pyr family of chemicals (the pyrtriazole series), developed anti-inflammatory SOCE inhibitors [82]. Aside from that, there were two compounds in this series that were surprisingly discovered to be SOCE activators, activating the channel at 198–236% and 142–197% entry at 10 µM in three distinct cell lines (Hek, Jurkat, and BV-2), respectively. Furthermore, the modest differences identified between the two compounds were due to the fact that 2T was more potent than the other. The impact of 2T was obvious at concentrations more than 0.3 µM, while the effect of 3G was visible at concentrations greater than 3 µM, with the effect of 3G increasing up to the maximum concentration (100 µM) tested [82].

### 3.4. IA65

IA65 or 4-((5-phenyl-4-(trifluoromethyl)-thiazol-2-yl)amino)benzoic acid has shown in recent studies to increase Orai1 activity in heterologous expression, as well as in vascular smooth muscle cells and skeletal muscle fibres [83]. In a concentration-dependent manner, IA65 also enhanced Ca^2+^-dependent inactivation (CDI) with an EC_50_ of 2 µM. IA65 has shown some selectivity by enhancing activity of ORAI1, while only slightly affecting Orai2 and 3. It is suggested that this Orai1 activator could be used as a tool to further understand the involvement of Orai1 in pathosphyisology [83]. Notably, IA65 has the reverse effect of Orai1 in that it suppresses Orai2 channel activation [72].

### 3.5. Inhibitors of ORAI/STIM Channel Complexes

SOCE is the predominant Ca^2+^ entry mechanism in mammalian cells and governs a number of cellular activities, including apoptosis, proliferation, motility, and death; therefore, inhibition of SOCE serves as potential drug target in several diseases and disorders [84].

### 3.6. Pyrazole Compounds: GSK-7975A and GSK-5503A

GSK-5503A and GSK-7975A are pyrazole derivatives that act as selective small molecule blockers of CRAC channels and TRPV6 channels (Table 2). GSK-7975A developed by GlaxoSmithKline inhibits I_CRAC_ channels in HEK293 cells and both Orai1 and Orai3 mediated I_CRAC_ in HEK293 cells with an IC_50_ of 4 μM (Table 2), respectively [85]. Studies have shown that the GSK compounds also potently block TRPV6 channels, as there are structural associations between CRAC and TRPV6 channels in the target site for the GSK compounds. This is while they have little or no influence on a number of other ion channels [86].

A separate study by Zhang et al. showed that GSK7975A largely inhibits Orai1 and Orai2 while it has a lower inhibitory effect against Orai3 [72]. As GSK7975A is unable to discriminate between Orai1 and Orai3 channels, as well as lacking the ability to block L-type Ca^2+^ and TRPV6 channels, the compound has a limited specificity as an Orai1 channel blocker [23].

GSK-7975A affects neither STIM1 oligomerization nor STIM1/Orai1 interaction [85]. These two pyrazole compounds could block STIM1-mediated Orai1 and Orai3 currents hypothetically via an allosteric effect on the Orai1 selectivity filter, having a gradual onset of action that effects STIM1-STIM1 oligomerization or STIM1-Orai1 coupling [85].

### 3.7. Compound 5J-4

Recently, Kim et al. noticed a novel family of compounds that block the activity of CRAC channels by using high-throughput chemical library screening targeting Orai1 [87]. The 5J-4 blocks the CRAC channel activity by preventing ion permeation, and this compound was more tolerated in vivo due to the removal of trichloride (-Cl3) [87]. The 5J-4 strongly blocked the SOCE in HeLa-O+S cells in addition to endogenous SOCE in TH17 cells [87]. The parent compound 5D had a half-maximum inhibitory concentration (IC_50_) of 807 nM and 195 nM (Table 2) for the peak and sustained levels of endogenous SOCE in primary murine effector T cells [87].

### 3.8. 2-APB and Its Analogues DPB162-AE and DPB163-AE

2-Aminoethyldiphenyl borate (2-APB) is nonselective Ca^2+^ entry blocker that has a biphasic effect on CRAC channel activation (Table 2). 2-APB at higher concentrations (20–100 μM, see Table 2) causes a transient activation of the CRAC channel, followed by a very potent inhibitory effect, which was observed even at low concentrations (1–10 μM), potentiating the CRAC channel activity [24,88]. The suggested mechanism of action for 2-APB is possibly through STIM1 multimerization, STIM1– Orai1 interaction, or on the Orai channel itself [31,89,90].

There have been studies on the mechanisms underlying the dual effects of 2-APB on SOCE, of which it has been reported that 2-APB might directly block STIM1 by enabling the connection between CC1 and SOAR. This is while it has been shown that 2-APB can also play a role in indirectly disrupting STIM1 functions by interfering the interaction between STIM1 and the mutant Orai1-V102C [91]. A number of research groups have also reported that 2-APB might directly gate and enlarge the pore diameter of Orai1 and Orai3 to control the SOCE pathway without interfering with STIM1 [2,92].

A number of studies have shown antineoplastic effects of 2-APB, making it a possible potent therapy for primary and metastatic cancers. Among others, when applied in high concentrations, 2-APB works as a blocker of SOCE, showing anticancer effects on various cancer types, including breast, ovarian, cervical, prostate, lung, melanoma, and glioblastoma [93,94,95,96,97].

Other studies have shown that 2-APB is effective in models of atherosclerosis, hypertension, and vascular calcification; however the effects are mostly attributed to the compound’s effects on TRP channels or IP3 receptor [23]. More studies are needed on the effects of 2-APB and its analogues on SOCE, as the mechanism of action is not fully understood.

The two 2-APB analogues, DPB162-AE and DPB163-AE, have quite similar chemical structures and only differ in the linker chain between their two diphenyl groups [90]. These two SOCE inhibitors are 100 times more effective than 2-APB, and they have the ability to block STIM1 clustering and inhibit the STIM1-triggered Orai1 and Orai2 activity by deactivating the STIM1-Orai activating region domain in STIM1.

DPB162-AE and DPB163-AE, like 2-APB, block the Orai1 currents and weaken the Orai2 currents. Nevertheless, in contrast to 2-APB, both fail to activate the Orai3 channels at higher concentrations in the absence of STIM1 [98]. However, the two mutants could both facilitate Orai3 currents at low concentration (~100 nM), and at high concentration (>300 nM) they transiently activated and then deactivated Orai3 currents. The DPB162-AE blocks the CRAC channel activity with IC_50_ = 200 nM and possibly acts directly on the coupling interface between the STIM1–Orai1 activating region (SOAR) and Orai1 [99,100]. Furthermore, studies have shown that DPB-162 could constantly block endogenous SOCE regardless of concentration strength, and when applying the greatest inhibitory effect on Ca^2+^ entry, little effect was applied on L-type Ca^2+^ channels, TRPC channels, or Ca^2+^ pumps [98,99].

To date, no studies have been reported on DPB compounds in regard to the specific SOCE inhibition and its effects on cancer treatment; however, DPB compounds are seen as potential anticancer drugs in the future.

### 3.9. Aspirin Metabolite Salicylate and Other NSAIDs

Nonsteroidal anti-inflammatory drugs (NSAIDs) such as aspirin and diclofenac are usually administered to relieve pain, fever, and inflammation; however, investigations have shown that this drug group likewise has cancer-protective effects, reducing the risk of a number of cancer types. A study by Núñez et al. showed that in colon cancer, NSAIDs could exert antiproliferative effects by inhibiting SOCE [101]. Burn et al. reported that patients suffering from Lynch syndrome are at high risk of developing CRC, and their study showed that aspirin protected against CRC [102]. Furthermore, the primary aspirin metabolite, salicylate, is regarded as a slight mitochondrial uncoupler that inhibits the proliferation of HT29 cells. A separate study has shown that applying ibuprofen together with indomethacin blocks COX-2, hindering STIM1-induced colorectal cancer development [103]. Muñoz et al. reported that NSAID drugs suppress vascular smooth muscle cell proliferation by facilitating the Ca^2+^-dependent inactivation of Orai channels, which mitochondria normally prevent [104]. Moreover, Muñoz et al. concluded that NSAIDs block Orai channels indirectly by preventing mitochondrial Ca^2+^ uptake, which facilitates the Ca^2+^-dependent inactivation of Orai channels [104].

### 3.10. SKF-96365

SKF-96365 is an antineoplastic drug, also known as a chemotherapy drug, and its effects are universal. This SOCE channel blocker is reportedly relatively nonselective, meaning it inhibits many other ion channels with similar potency. This compound is structurally different from other classic Ca^2+^ antagonists, which inhibits the entrance of Ca^2+^ by blocking its channels or affects its pools. The compound demonstrates selectivity as it inhibits receptor-mediated Ca^2+^ entry, causing a significant increase in [Ca^2+^]i while not affecting internal Ca^2+^ release in endothelial cells, platelets, or neutrophils [105].

Several studies have shown that SKF96365 inhibits cell proliferation by inducing cell apoptosis and cell cycle arrest in the G2/M phase in various types of cancers [60,72,100,106,107,108,109]. Furthermore, studies have shown that SKF 96354 could play a role in effectively blocking the metastasis of cancers by impairing the assembly and disassembly of focal adhesions of breast cancer cells, and block cell migration by deactivating nonmuscle myosin II, as well as decreasing actomyosin development and contractile force in cervical and non-small cell lung cancer (NSCLC) cells [100,110]. It has also been reported that by blocking TRPC channels in glioma cells, SKF 96365 could also be used as an adjuvant drug for radiotherapy. Analogues of SKF-96365 have been shown to have higher potency in blocking SOCE in B lymphocyte cells; however, further studies are needed to determine their specificity [111]. Azimi et al. found that SKF-96365 was active against Orai1-mediated Ca^2+^ influx; however, it was not selective with respect to inhibition of the Ca_V_2.2 channel using MDA-MB-231 breast cancer cells [83].

Despite the universal usage of SKF 96365 amid its antineoplastic effects, they are nonspecific, and further studies are needed to determine its specific mechanisms.

### 3.11. Mibefradil

Mibefradil is a T-type Ca^2+^ channel inhibitor was first used as a cardiovascular drug. However, lately it has been shown that Mibefradil blocks SOCE by inhibiting Orai channels in a dose-dependent and reversible manner, causing a significant inhibition of cell proliferation, inciting cell apoptosis and halting the cell cycle in the S and G2/M phases in HEK-293T-Rex cells [112].

### 3.12. 4-Choloro-3-ethylphenol (4-CEP) and Its Analogues 4-Choloro-m-cresol (4-CmC) and 4-Chlorophenol (4-CIP)

4-Choloro-3-ethylphenol (4-CEP) and its analogues 4-choloro-*m*-cresol (4-CmC) and 4-chlorophenol (4-CIP) all belong to a group of ryanodine receptor (RyR) agonists. The function of RyRs is to release the Ca^2+^ from intracellular store during excitation–contraction coupling in cardiac and skeletal muscle. A study by Zeng et al. has shown that 4-CEP blocked SOCE in rat L6 myoblasts and induced a significant Ca^2+^ release, while its derivatives 4-CmC and 4-CIP at the same concentration only showed a small increase and no effect, respectively [113]. The study also showed that 4-CEP can evoke Ca^2+^ release through RyR activation, but significantly blocks the store-operated Ca^2+^ influx in thapsigargin-treated, store-depleted STIM1/Orai1 cells. The results showed IC_50_ values for 4-CEP, 4-CmC, and 4-CIP of 203.6, 830.9, and 1437.1 µM, respectively (Table 2). Furthermore, the study suggested that 500 µM of 4-CEP is inhibited in HEK293 T-REx cells and three store-operated Orai currents are evoked by thapsigargin, namely Orai1/STIM1, Orai2/STIM1, and Orai3/STIM1, while the Orai currents are partially blocked by 4-CmC and 4-CIP using the same concentration. Testing the effects of these three RyR agonists on 2-APB-induced Orai3 currents using 500 µM of 4-CEP, 4-CmC and 4-CIP, the current was significantly blocked with potencies in the order of 4-CEP > 4-CmC > 4-CIP. Whole-cell recordings of Orai3 showed that the current was induced by 50 µM of 2-APB and was blocked by 4-CEP with an IC_50_ value of 71.0 ± 4.2 µM (Table 2). This IC_50_ value is lower compared to other non-selective store-operated channel blockers, including 2-APB, SKF96365, and BTP2 [106,114,115]. Zeng et al. also suggested the action site for 4-CEP, as intracellular application of 4-CEP did not block the Orai3 channels, although the extracellular perfusion blocked the current [114].

### 3.13. Lanthanides

At low concentrations, lanthanides such as lanthanum and gadolinium (Gd^3+^) block CRAC channels and micromolar Gd^3+^ inhibits Ca^2+^ influx, which is often seen as evidence for store-operated Ca^2+^ entry. Lathanides also play a role in many other ion channels, meaning that Gd^3+^ even at submicromolar concentrations are not considered a specific CRAC channel inhibitor [116]. Lathanides can inhibit SOCE at low concentrations (<1 μM). Studies on Orai channels have shown that lathanides act by access of Ca^2+^ ions to the selectivity filter and pore [117]. While at concentrations of more than 100 μM, lathanides begin to inhibit plasma membrane Ca^2+^ATPase (PMCA), and at concentrations above 1 mM they completely block the activity, making it seem as if the cytoplasm is isolated or “insulated” from the extracellular space. At this state, the “insulation” is stopping both the entry and efflux of Ca^2+^ ions, creating an opportunity for investigations of complex intracellular calcium signalling events unassisted by constituents in the extracellular space [77].

### 3.14. Bistrifluoromethyl-Pyrazole Derivative (BTP-2) or Pyrazole Derivatives

Bistrifluoromethyl-pyrazole derivative, known as Pyr2, BTP2, or YM-58483, was initially reported as an SOCE inhibitor, reducing IL-2 production and NFAT dephosphorylation in Jurkat cells without altering the T-cell receptor signal transduction pathway [115]. BTP2 reportedly inhibits cytokine production and proliferation in electrically non-excitable T cells [118]. Studies have shown that YM-58483 does not cross-react with other ion channels, including voltage-operated Ca^2+^ entry, chloride (Cl^−^) channels, or potassium (K^+^) channels [115,119,120]. The exact molecular mechanism of this compound is unknown; nevertheless, YM-58483/BTP2 suppresses a number of ion channels such as CRAC, TRPC3, and TRPC5, as well as facilitating the TRPM4 channel [115,119,120]. YM-58483 is also a potent blocker of thapsigargin-induced SOCE Ca^2+^ influx. In a study by Azimi et al. using MDA-MB-231 breast cancer cells, YM-58483 inhibited SOCE mediated by Orai1 with an IC_50_ value of 2.8 µM, while the compound was inactive against TRPV1, TRPM8, and Ca_V_2.2 at concentrations up to 100 µM (Table 2) [121].

He et al. studied 3,5-bis(trifluoromethyl)pyrazole derivative BTP2 and its effect on SOCE activation in various cell types and assessed its modification of TRPC3, TRPC5, and TRPV6 with an aim to find any link between the function of the TRP channel and SOCE [119]. Using human embryonic kidney (HEK) 293 cells, chicken DT40 B cell line, and A7r5 embryonic rat VSMCs, the group found that BTP2 inhibited SOCE within 10 min with an IC_50_ value of 0.1–0.3 µM. BTP also blocked the T3-65 clonal HEK293 cell line, stably expressing TRPC3 channels and TRPC3-mediated Sr^2+^-entry activated by muscarinic receptors with an IC_50_ value of <0.3 µM. The study also showed that BTP2 inhibited direct activation of TRPC3 channels by diacylglycerol, with an IC_50_ value of about 0.3 µM. BTP2 also blocked the whole-cell carbachol-induced TRPC3 current by 3 µM. Recordings of the single TRPC3 channel showed persistent short openings, indicating that BTP2 diminishes the channel’s open probability rather than its pore properties. In the case of TRPC5 channels transiently expressed in HEK293 cells, BTP2 blocked in the same range as TRPC3. This is while BTP2 did not affect the function of the highly Ca^2+^-selective TRPV6 channel at all, despite having as many channel properties as SOCs. He et al. concluded with these results that there is a strong functional relation between the operation of the expressed TRPC channel and endogenous SOC activity [119].

### 3.15. Synta-66

Synta-66 (N-(2′,5′-dimethoxy-[1,1′-biphenyl]-4-yl)-3-fluoroisonicotinamide) is a selective CRAC channel inhibitor developed by Synta Pharmaceuticals (Table 1). Synta-66 inhibits I_CRAC_ in rat basophilic leukaemia (RBL) cells with an IC_50_ value of 1.4 μM [122], while not affecting plasma membrane Ca^2+^ ATPase pump and inwardly rectifying K^+^ channels.

A study by Li et al. showed that Synta-66 is a potent SOCE inhibitor in VSMCs, which are isolated from human saphenous veins [123]. The results showed a significant reduction in VSMC migration, with an IC_50_ of around 26 nM [123]. Despite various studies of Synta-66, the mechanism of action for the compound has not yet been fully clarified. Furthermore, due its poor aqueous solubility, Synta-66 probably will not be suitable for therapies [23]. It is worth noting that Synta66 inhibited Orai1 channel activity while potentiating Orai2, and had no impact on Orai3 [72].

### 3.16. RO2959

The CRAC inhibitor RO2959 (Carboxyamidotriazole; 2,6-Difluoro-N-tetrahydro-pyridin-3-yl]-pyrazin-2-yl)-benzamide) was characterized by Chen G. et al., and potently blocks human T-cell activation and effector functions (Table 2) [124]. In more detail, by using both electrophysiological and calcium-based fluorescence measurements, this group showed that the RO2959 was able to block the IP3-dependent current in CRAC-expressing RBL-2H3 cells and CHO cells stably expressing human Orai1 and Stim1, as well as SOCE in human primary CD4(+) T cells triggered by either TCR stimulation or thapsigargin treatment [124]. Originally, RO2959 was synthesized by Synta Pharmaceutical and has been identified as a novel, potent, and selective I_CRAC_ inhibitor by Roche [90]. The I_CRAC_ inhibitor is efficient at concentrations of nanomolars and demonstrates superior selectivity for Orai1 inhibition compared to Orai2 and Orai3. In a study by Chen et al., RO2959 at a 3 µM (Table 2) concentration inhibited the 5-hydroxytryptamine receptor 2B (5-HT2B) by 87% and the peripheral benzodiazepine (BZD) receptors by 89%. Despite this progress, the mechanism of action of RO2959 has yet to be determined [124].

### 3.17. CM4620, CM2489, CM3457, CM128 (CalciMedica Series)

CM4620 is a small molecule Orai1 inhibitor with a IC_50_ = 119 nM (Table 2) and displaying a weaker degree of effectiveness when used against Orai2 at 895 nM [125]. CM2489 and CM3457 are small molecule inhibitors of CRAC channels developed by CalciMedica (Table 2). The structure of CM2489 has not been revealed yet; however, it is the first CRAC channel blocker to be tested in humans for treating moderate-to-severe plaque psoriasis, and has completed Phase I clinical trials, although it was discontinued. This is while the efficacy of CM2489 as an inhibitor of different Orai isoforms and its mechanism of action have not been identified yet. The structure and mechanism of CM3457 remains unclear; although it has been shown that the compound inhibits T-cell proliferation, Th1, Th2, and Th17 induced cytokine production and mast cell degranulation in vitro. Studies on rats have revealed that orally administered CM3457 was effective in treating joint inflammation in a rat collagen-induced arthritis model, and the compound had an inhibitory effect on lung inflammation and eosinophilia in an ovalbumin-induced rat asthma model [126].

### 3.18. AnCoA4

AnCoA4 was discovered during a mass screening of 12,000 compounds, in which hits are found when small molecule compounds that bound to Orai1 and/or STIM1 in a microarray system containing minimal functional domains of STIM1and Orai1. In the study, AnCOA4 showed 80% inhibition of SOCE (HEK-293 cells, coexpressing STIM1/Orai1) at 20 µM [127]. Both in vitro and in vivo studies have shown that AnCoA4 can block CRACs and reduce the amount of T-cell activation. It carries this out by lowering the amount of Orai1 that is recruited into puncta and by inhibiting the activity of the constitutively active Orai1 V102C channels. Notably, this effect is completely independent of STIM1 [3].

### 3.19. ML-9

ML-9 (1-(5-chloronaphthalene-1-sulfonyl)-1H-hexahydro-1,4-diazepine), is a specific myosin light chain kinase (MLCK) inhibitor that reversibly inhibits SOCE with an IC_50_ value of 10 μM (Table 2). A study by Smyth et al. showed that ML-9 both prevented SOCE development and blocked SOCE following its activation [128]. It was also revealed that the effect of Ml-9 was reversible, meaning that the I_CRAC_ current was restored after the compound was washed out [128,129]. According to a number of studies, ML-9 is one of a small set of pharmacological compounds that appear to have STIM1 as their target rather than Orai1, showing that STIM1 activation can be independently targeted to control the activity of CRAC Channel [79]. It is yet not determined where the action site of ML-9 on STIM1 is located.

### 3.20. JPIII

Recently, Bartoli et al. developed a new analogue of Synta-66 as a potent Orai1 inhibitor with superior pharmacokinetics over Synta-66 [130]. Moreover, in vivo, JPIII exhibited great effectiveness with no noticeable adverse effects in mouse models [130]. Notably, in HEK293 cells, JPIII demonstrated substantial nanomolar (IC_50_ = 399 nM) inhibitory effects of SOCE (Table 2). Furthermore, JPIII exhibited excellent selectivity towards Orai1 and had no effect on the activity of Orai3, TRPC4, TRPC5, TRPC6, TRPM2, or hERG channels [130].

### 3.21. Pyrtriazole Compound 39

SOCE inhibitors with anti-inflammatory properties that are derived from the Pyr family have been developed using the pyrtriazole series [82]. In HEK293 cells, lead Pyrtriazole compound (39) inhibited SOCE with an IC_50_ = 4.4 µM (Table 2) and was shown to be selective for SOCE over TRPM8, TRPV1, and voltage-gated Ca^2+^ channels, despite an analogue activating TRPV1. Moreover, Pyrtriazole 39 was tested in a mouse model of acute pancreatitis and was shown to decrease oedema, inflammation, and apoptosis, all of which are symptoms of the disease [82].

### 3.22. RP3128 and RP4010

Rhizen Pharmaceuticals has created SOCE inhibitors for cancer therapy, and two of them have progressed to the stage of clinical trials. In a guinea pig model of asthma, RP3128 was shown to be orally active and efficacious. It was also enrolled in a Phase I dosage escalation safety trial; however, it was later discontinued [131,132,133]. RP4010 has been explored for the treatment of oesophageal squamous cell carcinoma and has proven to be potent in various cancer cell lines as well as in xenograft animal models of the disease [3]. It needed approximately two hours to display peak inhibition of SOCE, indicating that it might have a less direct effect on the channel than immediately inhibiting Orai1 as previously thought [3]. It was enrolled in Phase I safety trials for the treatment of relapsed non-Hodgkin’s lymphoma; however the trial was stopped due to the failure of pharmacokinetic (PK) and safety considerations [134].

Only very recently, in duct cells, both in vitro and in vivo, Pallagi et al. discovered the effects of another ORAI1 inhibitor, CM5480 (CalciMedica Inc.), utilizing alcohol- or bile-induced acute pancreatitis (AP) [135]. In a cerulein-induced experimental animal model of AP, CM5480 dramatically reduced pancreatic damage while inhibiting Ca^2+^ influx in duct cells more slowly than GSK7975A [135].

A recent study regarding TAM, which involved wild-type and STIM1 p.I115F mice, showed that SOCE inhibitor CIC-39Na, restored platelet numbers and reverted the characteristic abnormal bleeding. CIC-39Na might be a pharmacological treatment strategy for thrombocytopenia in TAM patients [136]. Moreover, it is worth mentioning that 1,2,4-Oxadiazole-Bearing Pyrazoles was described as new SOCE inhibitor and as metabolically stable modulators of SOCE by [137]. The role of STIM2 in melanoma is still poorly understood; however, a study by Stanisz et al. described that STIM2 might act both as a tumour suppressor in highly proliferative cells and a tumour promoter in specific invasive cancers in which increased basal [Ca^2+^]i results in a more invasive phenotype [138].

## 4. Conclusions

A spike in cytosolic calcium concentration regulates biological responses ranging from sperm motility and egg fertilization to cell death via apoptosis and necrosis. Moreover, cytosolic Ca^2+^ controls a variety of processes occurring during the life–death cycle, including exocytosis, energy generation, gene transcription, and cell motility. In 1986, the concept of store-operated Ca^2+^ entry was introduced. Furthermore, over the past three decades, a large and growing body of literature has investigated the physiological and pathological role of the Orai/STIM channels in different cell types and tissues. These channels are distinctive Ca^+2^ signal mediators in most cell types, and their regulation and function are unmatched compared to other ion channels. They function with high selectivity for Ca^+2^ and remarkably mediate both short-term Ca^+2^ homeostasis and long-term Ca^+2^ signals [140].

It has been demonstrated that the Orai/STIM channels are involved in immunodeficiency, myopathy, tubular aggregate, Stormorken syndrome, York platelet syndrome, cardiovascular and metabolic disorders, cancers, kidney diseases, and other diseases and disorders [33,36,37,44,46,60,73,75].

SOCE signals are vital for cellular regulation and many cellular functions. The SOCE pathway mediated by CRAC channels regulates various physiological functions. It has been determined that ORAI1 protein is crucial for native mammalian CRAC channels, while ORAI2 and ORAI3 still remain unclear. While the crystal structure of the sole ORAI isoform in drosophilia has been determined, the relative contributions of ORAI1, ORAI2, and ORAI3 to the stoichiometry of native CRAC channels remain unresolved. Having more insight in the interaction between STIM and ORAI on the molecular level and the signal transduction that leads to channel activity would play a crucial role in further comprehending SOCE dynamics and its role in vital cellular functions, and furthermore, finding specific targets in the treatment of diseases and disorders.

Taken together, the Orai/STIM channels are crucial protein complexes as drug targets. Moreover, the absence of trustworthy antibodies, channel potentiators, or blockers that are capable of distinguishing different Orai isoforms has impeded our knowledge of the physiological roles. On that account, the next step in the fields of Orai/STIM channels will be the identification of potent, selective, and subtype-specific small molecules as agents to treat human diseases and disorders for patient benefit.

## Figures and Tables

**Table 1:** Orai/STIM channel complexes in human diseases.

Diseases	Subunit Combination	Reference(s)
Immunodeficiency	Orai1/STIM1	[33]
Myopathy	Orai1/STIM1	[37]
Tubular aggregate myopathy	Orai1/STIM1	[36]
Stormorken syndrome	Orai1/STIM1	[44]
York platelet syndrome	Orai1/STIM1	[46]
Cardiovascular and metabolic disorders	Oria1/STIM1	[75]
Cancers	Oria1/STIM1 ORAI3 (lung cancer) STIM2 (tumour growth)	[60] [68] [63]
Kidney	Orai1	[73]

**Table 2:** Selected Orai-STIM channel blockers.

Name	IC_50_	Cell Types	Reference
GSK-5503A & GSK-7975A	4 μM	HEK-293	[85]
5J-4	807 nM (peak) 195 nM (sustained)	Primary murine effector T TH17 HeLa-O + S cells	[87]
DPB162-AE	200 nM	HEK-293	[99]
4-CEP	203.6 µM	HEK-293	[113]
4-CmC	830.9 1 µM	HEK-293	[113]
4-CIP	1437.1 µM	HEK-293	[113]
SKF96365	12 µM	T leukaemic	[139]
BTP2 (YM-58483)	2.8 µM	MDA-MB-231 breast cancer cells	[121]
Synta-66	1.4 μM	Rat basophilic leukaemia (RBL)	[122]
ML-9	10 μM	HEK-293	[128,129]
JPIII	399 nM	HEK-293	[130]
Pyr 39	4.4 μM	HEK-293	[82]
RO2959	265 nM	CD4+T lymphocytes	[124]

## Data Availability

Data sharing not applicable.
